# Household barriers and facilitators to healthy eating in a U.S. census-representative sample of the general population and a low-income sample: a cross-sectional survey

**DOI:** 10.3389/fpubh.2025.1648218

**Published:** 2025-08-13

**Authors:** Kayla E. Tate, Angel Bassett, Heather Gavras, Cheryl D. Toner, Kristina S. Petersen

**Affiliations:** ^1^Department of Nutritional Sciences, The Pennsylvania State University, University Park, PA, United States; ^2^American Heart Association, Dallas, TX, United States

**Keywords:** food choice, healthy eating, barriers & facilitators, diet quality, food purchasing practices

## Abstract

**Introduction:**

The present study aimed to quantify differences in barriers and facilitators to healthy eating experienced in a U.S. census-representative sample of the general population and a sample of low-income individuals.

**Methods:**

A cross-sectional survey was administered to U.S. adults. Barriers and facilitators to healthy eating were assessed with survey questions pertaining to important and influential attributes for food purchasing, attitudes about food purchasing, and barriers and facilitators to choosing healthy foods. Two samples were recruited: (1) a general sample that was census-representative for age, income, gender, and geographic region approximated from the 2022 US census data (*n* = 1,182); (2) a low-income sample that reported an income of less than $34,000 per year and participating in federal food or healthcare assistance programs (*n* = 506). Differences between samples for barriers and facilitators to healthy eating were assessed using chi-square tests for proportions.

**Results:**

Taste and cost were identified as key barriers to healthy eating across all survey questions, and nutritional value of foods was not found to be a priority. Facilitators to healthy eating included having access to budget friendly and good tasting recipes and preparing grocery lists in advance.

**Discussion:**

Future interventions seeking to improve diet quality may benefit from emphasizing flavor and taste as necessary components of healthy eating.

## 1 Introduction

Cardiometabolic diseases (CMDs) are among the leading causes of death globally and in the US (1), and poor diet quality is a key modifiable risk factor for CMDs (2). Poor diet quality is defined by inadequate intakes of nutrient-dense foods and overconsumption of saturated fat, added sugars and/or sodium (3). While diet quality is sub-optimal across income, race and ethnicity, gender, and age groups in the U.S. (4), disparities in diet quality exist by income (5), race and ethnicity (6), gender identity (7), and age (8). Given these well-established disparities in diet quality, it is important to investigate the unique barriers and facilitators to healthy eating for each demographic group. Understanding these barriers and facilitators may help to inform programs and policies to facilitate a food system that promotes adequate diet quality for all (9).

To understand barriers and facilitators to healthy eating, it is first necessary to consider the determinates of food choice. Food choice is highly complex and is influenced by a wide variety of factors both within and outside of individual control. Determinants of food choice can operate across multiple levels of influence, ranging from systemic and structural factors, including policies and the resulting food environments, to individual factors, such as the physiological experience of hunger (10). A broad framework characterizing the determinates of food choice suggests that factors including the features of foods (i.e., taste, nutritional value, availability), differences between individuals (i.e., nutrition knowledge, preferences, and attitudes), and the effects of societal-level features (i.e., food prices, socioeconomic status) are key determinates driving food choices (11). Determinates of food choice may be barriers or facilitators to choosing and consuming healthy foods. Much work to date has been done to characterize barriers and facilitators to healthy eating in a wide variety of populations, including healthcare workers (12), cancer survivors (13), college students (14), low-income populations (15), and others. Constructs such as food costs, flavor/taste, preparation time, and lack of available healthy options coupled with an abundance of unhealthy options, have all been identified as barriers to healthy eating in several populations (12, 16, 17). Conversely, constructs such as nutrition knowledge, skills, and social support have been identified as facilitators to healthy eating (18).

Many of the prior studies investigating barriers and facilitators to healthy eating have been conducted within specific communities, and may lack generalizability to the US population as a whole, or may lack generalizability to the low-income populations at greatest risk of poor diet quality. Furthermore, much of the evidence base is comprised of studies that have qualitatively investigated barriers and facilitators to healthy eating (15, 17, 19). While qualitative methods provide deeply nuanced perspectives and insights within specific populations, they do not allow for quantitative comparisons of demographic groups within or between populations. As such, there is a current gap in the understanding of what proportion of the US population may be experiencing specific barriers or facilitators for healthy eating, and how these proportions may differ between demographic groups. In addition, it is also not clear how high- and low-income populations differ in terms of the relative proportion of each group facing specific barriers and facilitators to healthy eating. Thus, the present study aims to explore the differences in the proportions of barriers and facilitators to healthy eating reported in a US census-representative sample of the general population and in a sample of low-income individuals. This study also aims to explore the proportions of barriers and facilitators to healthy eating reported by different age groups, races and ethnicities, and gender identities within each sample. Understanding the barriers and facilitators to healthy eating (i.e., the consumption of a high-quality diet) and how they differ across income, race and ethnicity, gender identity, and age groups is expected to assist with the development of interventions for improving diet quality.

## 2 Methods

### 2.1 Study overview and participants

A cross-sectional survey of U.S. adults was conducted. Participants were recruited by a market research company (Hanover Research, VA) from a pre-established market research panel between May and June 2023. Participants were asked to complete a 10–12 min online survey provided in English about barriers and facilitators to healthy eating. To prevent duplicate responses or bots, IP addresses were tracked and hidden questions designed to catch bots were included in the survey. The survey began with items assessing eligibility criteria, and if participants were determined ineligible, the survey ended. Participants were eligible if they were at least 18 years old and were the primary or shared decision maker for household food choices. Participants were excluded if they worked in an industry that was believed to influence responses, which is standard practice in market research (20). Industries that were exclusionary were healthcare, nutrition, advertising, public relations, marketing, or market research jobs. Two participant samples were recruited: (1) a general sample (GEN) that was approximately census-representative for age, income, gender, and U.S. geographic region approximated from the 2022 US census data (21) (n = 1,182); (2) a low-income sample (LI) that reported an income of less than $34,000 per year and reported participating in federal food or healthcare assistance programs (n = 506). $34,000 per year was selected as the threshold for defining low-income because this number approximately aligns with census poverty thresholds for a family of four (22). Participants were compensated $2.80 and $4.50 for the GEN and LI samples, respectively. A larger compensation was provided to the LI sample to facilitate participation given the lower response rates for this demographic within the panel system. Study materials were reviewed by Advarra IRB (Columbia, MD) and classified as exempt, under federal regulations [45 CFR 46.104(d)(2)] (23), from IRB oversight.

### 2.2 Measures

Participants were asked to report demographic characteristics including their race and ethnicity, gender identity, and age group. Race and ethnicity responses included White or Caucasian, Black or African American, Hispanic, Asian, American Indian or Alaska Native, Native Hawaiian or another Pacific Islander, or prefer not to share. Those selecting American Indian or Alaska Native, Native Hawaiian or Pacific Islander, or prefer not to share were grouped together due to low response rates in these categories. Any participants selecting more than one race or ethnicity were categorized as multiracial. Gender identity responses included male, female, non-binary and not listed/prefer to self-describe. Those identifying as non-binary or who indicated a preference to self-describe were grouped into one category due to low response rates in each category. Age was analyzed as a categorical variable (18–24, 25–34, 35–44, 45–54 and ≥55 years) to allow for subgroup analyses. Additional demographic and descriptive questions included level of education (e.g., some high school, high school diploma/GED, some college or post-secondary technical training, 2-year degree, 4-year degree, some graduate school, graduate degree), marital status (e.g., single/never married, married or living as married, separated or divorced, widowed), number of people in the household (e.g., 1 person, 2 people, 3 people, 4 people, 5 or more people), residential area type (e.g., urban area, suburban area, rural area), perceived diet quality (e.g., poor fair, good, very good, excellent) and amount of money spent on groceries per month (e.g., less than $25, $25–$49, $50–$74, $75 to $100, more than $100). For these descriptive questions, participants were presented with a number of responses and instructed to select one option.

Due to a paucity of survey instruments in alignment with the study aims, a novel survey instrument was developed in collaboration with Hanover Research. This instrument was developed through conducting non-systematic literature reviews to identify common barriers and facilitators to healthy eating and incorporating identified barriers into multiple survey domains and items. Expert opinion from authors AB, HG, and CD was also utilized to develop the survey. Barriers and facilitators to healthy eating were assessed with several survey domains, including important attributes for food purchasing, influential attributes for food purchasing, attitudes about food purchasing, healthy eating behaviors, and barriers and facilitators to choosing healthy foods in general (Table 1).

**Table 1:** Descriptions of survey domains, domain items, and item response options.

Survey domain	Survey question	Domain items or response options
Attitudes about food purchasing	To what extent do you agree with each of the following statements? (Options: *Strongly disagree, disagree, neither agree nor disagree, agree, strongly agree*).	I am willing to pay more for a food product if it satisfies my personal tastes
		I often look at product labels for information about origin and sourcing practices
		I prefer to buy local products when possible
		I tend to avoid eating carbs and think they are unhealthy
		I tend to seek out social and emotional experiences with food
		I usually select food products according to brand
		I usually select food products according to nutrition (e.g., calories, salt) and health properties
		I usually select food products according to price
		The health and nutritional information shown on the label is important when choosing a food product
		When buying a food product, I am heavily influenced by product sales or discounts
		When buying a food product, I prefer a long shelf life
		When buying a food product, I prefer a lower cost
Important attributes for food purchasing	How important are the following attributes when purchasing food for your household? (Options: *Not at all important, slightly important, moderately important, very important, extremely important*).	Price Flavor/Taste High quality/premium food Nutritional value Ingredients Easy to prepare Organic/Natural Availability Convenience Serving size Shelf-life Brand Requires less time to prepare Dietary philosophy No/low preservatives added Environmentally friendly
	What would you say is the most important attribute when purchasing food for your household? (Options: Any of the above items rated as “very important” or “extremely important” appeared in this list)	
Influential attributes for food purchasing	How influential are each of the following when purchasing food for your household? (Options: *Not at all influential, slightly influential, moderately influential, very influential, extremely influential*).	Cooking skill/ability
		Cultural traditions
		Environmental sustainability
		Feeling hungry
		Health benefits
		I have a coupon/discount
		It is on sale
		My current mood
		Previous experiences
		Religious practices
		Running low/ran out
		Short time to prepare
		Specific diet requirements
		Supporting local business
Barriers to consuming healthy foods	What prevents you from making at least half of your grains whole grains? *Select all that apply.*	Taste preferences
		Cost
		I do not understand the difference between whole and refined grains
		I did not know this was recommended
		I’m not sure how to use/prepare
		Dietary restrictions
		Cultural/Religious preferences
		I do not care/try
		I do not know/unsure
		Other
	What prevents you from choosing healthy sources of protein? *Select all that apply*.	Cost
		Taste preferences
		I did not know this was recommended
		I’m not sure how to use/prepare
		Dietary restrictions
		Cultural/Religious preferences
		I do not understand which are healthy choices
		I do not care/try
		I do not know/unsure
		Other
		None of the above
	Which of the following reasons, if any, prevent you from selecting healthier foods? *Select all that apply.*	Price is too high
		Does not taste as good
		Shelf life too short
		Preparation time is too long
		Not as available
		Serving sizes too small
		I do not always have the right cooking tools or equipment
		Nothing prevents me from selecting healthier foods
		Other
Facilitators to consuming healthy foods	Which of the following, if any, have helped support you in selecting healthier foods? *Select all that apply.*	Preparing grocery list in advance
		Good-tasting recipes
		Budget-friendly recipes
		Meal-prepping in advance
		Watching cooking videos
		Access to the right cooking tools or equipment
		Following health blogs/influencers
		Recommendations from organizations
		Consulting a dietician/nutritionist
		Nothing supports me in selecting healthier foods
		Other

Survey domains assessing important and influential attributes for food purchasing and attitudes about food purchasing were each presented as a question (i.e., “How influential/important are each of the following when purchasing food for your household?”) with several items to be rated individually on a 5-point Likert type scale. The important attributes for food purchasing survey domain predominately included items relevant to specific food products (i.e., “brand,” “serving size,” “shelf life” etc.). If participants selected “important” or “very important” for an item under the important attribute’s domain, the item was presented as a response option in the next survey question that asked participants to select the single most important attribute for food purchasing. The influential attributes for food purchasing survey domain included items relevant to how a consumer may make decisions about food purchases, such as consumer beliefs or values (i.e., “health benefits,” “cultural traditions,” “supporting local business” etc.) or current state of the consumer (e.g., “feeling hungry,” “my current mood,” etc.). The attitudes about food purchasing domain asked participants to what extent they agreed with various statements about food purchasing with responses following a 5-point Likert scale. The healthy eating behaviors domain included items relevant for understanding consumer perceptions of and purchasing behaviors, with a focus on whole grains and healthy protein foods as under-consumed food categories in the U.S. The barriers and facilitators to choosing healthy foods in general domain included items relevant for understanding consumer perceptions of barriers and facilitators to choosing healthy foods. The healthy eating behaviors and the barriers and facilitators to healthy eating domains were each presented as multiple items (i.e., “Which of the following, if any, have helped support you in selecting healthier foods?” etc.) with a list of several response options (i.e., “preparing grocery lists in advance” etc.) where participants were instructed to select all responses that applied for each item. See Table 1 for a description of each survey domain, including items with each domain and the response options for each item.

### 2.3 Statistical analyses

Differences between samples for each survey domain, including descriptive and demographic survey questions, were assessed with chi-square tests for proportions. For survey domains with multiple items (i.e., Likert and Likert-type scales) or multiple response options within each item (i.e., barriers and facilitators to healthy eating), to reduce the total number of statistical tests conducted, only the three items or responses selected by the largest proportions of the samples were assessed for statistical differences. For Likert and Likert-type survey questions (e.g., attitudes, important and influential attributes for food purchasing), responses for each item within the survey domains were converted to binary variables such that one category indicated agreement with or importance/influence of the item, and the other category indicated neutrality/disagreement with or unimportance/noninfluence of the item. Next, the three items within each domain that had the largest proportion of affirmative responses (i.e., agreement or importance/influence) within each sample were identified. Chi-square tests for proportions were used to assess differences in the proportions between each sample for these items. Binary variables were used for the Chi-square tests to assist with meeting cell-count assumptions. For all statistical comparisons, Fisher’s exact tests were used if expected cell count assumptions were not met for chi-square tests. Results were considered statistically significant at *α* < 0.01 to reduce type I error. Response frequencies for each variable set were also cross-tabulated by sample, age group, race and ethnicity, and gender identity to identify trends between demographics within each sample.

## 3 Results

### 3.1 Sample description

In total, 7,546 individuals from the market research panel entered the survey tool, 4,195 were determined to be eligible, and 1,688 completed the survey and were included in the data analysis (GEN: n = 1,182; LI: n = 506). The mean age was similar across the samples (GEN: 48 ± 17.6 y; LI: 48 ± 17.6 y) and both samples were predominately White (Table 2). Samples differed with regard to gender identity, race and ethnicity, level of education, marital status, number of people in the household, residential area type, income level, and benefits, money spent on groceries, and perceived diet quality.

**Table 2:** Demographic characteristics of the survey respondents by sample.

Characteristic	General sample *n* = 1,182	Low-income sample *n* = 506	*P*-value^a^
Gender			
Male	583 (49.32)	184 (36.36)	<0.001
Female	597 (50.51)	316 (62.54)	<0.001
Non-binary	2 (0.17)	6 (1.19)	0.005
Age (years)			
18–24	141 (11.93)	72 (14.23)	0.192
25–34	192 (16.24)	64 (12.65)	0.059
35–44	233 (19.71)	80 (15.81)	0.056
45–54	184 (15.57)	92 (18.18)	0.183
55+	432 (36.55)	198 (39.13)	0.315
Race and ethnicity^b^			
White	854 (72.87)	367 (72.67)	0.907
Black	138 (11.77)	69 (13.66)	0.260
Hispanic	62 (5.29)	16 (3.17)	0.062
Asian	59 (5.03)	9 (1.78)	0.002
Multi-racial	51 (4.35)	38 (7.52)	0.007
Other	18 (1.52)	7 (1.38)	0.828
Education			
Some high school	24 (2.03)	38 (7.51)	<0.001
High school diploma/GED	225 (19.04)	182 (35.97)	<0.001
Some college/ technical training	279 (23.60)	154 (30.43)	0.003
2-year degree	110 (9.31)	51 (10.08)	0.620
4-year degree	302 (25.55)	53 (10.47)	<0.001
Some graduate school	37 (3.13)	10 (1.98)	0.187
Graduate degree	202 (17.09)	18 (3.56)	<0.001
Marital status			
Single, never married	338 (28.60)	207 (50.91)	<0.001
Married or living as married	671 (56.77)	133 (26.28)	<0.001
Separated or divorced	128 (10.83)	120 (23.72)	<0.001
Widowed	43 (3.64)	45 (8.89)	<0.001
Number of people in household			
1 person	224 (18.95)	189 (37.35)	<0.001
2 people	433 (36.63)	152 (30.04)	0.009
3 people	224 (18.95)	76 (15.02)	0.053
4 people	182 (15.40)	43 (8.50)	<0.001
5 or more people	113 (9.56)	45 (8.89)	0.667
Which area do you live			
Urban area	343 (29.02)	172 (33.99)	0.042
Suburban area	605 (51.18)	188 (37.15)	<0.001
Rural area	227 (19.20)	143 (28.26)	<0.001
Income			
$0 – $34,999	97 (8.1)	506 (100)	<0.001
$35,000 to $49,999	269 (22.76)	0 (0)	<0.001
$50,000 to $74,999	235 (19.88)	0 (0)	<0.001
$75,000 to $99,999	154 (13.03)	0 (0)	<0.001
$100,000 to $124,999	132 (11.17)	0 (0)	<0.001
$125,000 to $149,999	101 (8.54)	0 (0)	<0.001
$150,000 to $174,999	66 (5.58)	0 (0)	<0.001
$175,000 to $199,999	38 (3.21)	0 (0)	<0.001
$200,000+	63 (5.33)	0 (0)	<0.001
Prefer not to say	27 (2.28)	0 (0)	<0.001
Benefits			
Medicare	346 (29.27)	220 (43.48)	<0.001
Social Security	324 (27.41)	171 (33.79)	0.008
SNAP	173 (14.64)	290 (57.31)	<0.001
Medicaid	190 (16.07)	252 (49.80)	<0.001
Social Security Disability Insurance	85 (7.19)	92 (18.18)	<0.001
Supplemental Security Income	59 (4.99)	72 (14.23)	<0.001
Free or reduced-price school meals	68 (5.75)	48 (9.49)	0.006
WIC	36 (3.05)	32 (6.32)	0.002
Children’s health insurance program	53 (4.48)	12 (2.37)	0.039
Unsure	53 (4.48)	0 (100)	<0.001
Other	14 (1.18)	9 (1.78)	0.335
None per household	495 (41.88)	0 (100)	<0.001
How would you rate your eating habits?			
Poor	38 (3.21)	38 (7.51)	<0.001
Fair	219 (18.53)	159 (31.42)	<0.001
Good	503 (42.55)	214 (42.29)	0.920
Very good	317 (26.82)	80 (15.81)	<0.001
Excellent	105 (8.88)	15 (2.96)	<0.001
How would you rate the nutritional quality of the food you regularly purchase and consume?			
Poor	33 (2.79)	25 (4.94)	0.026
Fair	183 (15.48)	148 (29.25)	<0.001
Good	495 (41.88)	223 (44.07)	0.404
Very good	353 (29.86)	89 (17.59)	<0.001
Excellent	118 (9.98)	21 (4.15)	<0.001
Money spent on food per week			
<$25	41 (3.47)	47 (9.29)	<0.001
$25 – $49	125 (10.58)	111 (21.94)	<0.001
$50 – $74	240 (20.30)	132 (26.09)	0.009
$75 – $100	355 (30.03)	127 (25.10)	0.040
More than $100	421 (35.62)	89 (17.59)	<0.001
In the last 12 months, how often did the food you buy not last, and did you not have money to buy more?			
Always	79 (6.68)	50 (9.88)	0.024
Most of the time	142 (12.01)	95 (18.77)	<0.001
Sometimes	265 (22.42)	172 (33.99)	<0.001
Rarely	227 (19.20)	98 (19.37)	0.938
Never	460 (38.92)	89 (17.59)	<0.001
Prefer not to answer	9 (0.76)	2 (0.40)	0.392
In the last 12 months, how often have you skipped a meal because money was short?			
Daily	58 (4.91)	38 (7.51)	0.034
A few times a week	124 (10.49)	103 (20.36)	<0.001
Once a week	69 (5.84)	45 (8.89)	0.022
Once every 2 to 3 weeks	88 (7.45)	54 (10.67)	0.029
Once a month	68 (5.75)	32 (6.32)	0.649
Less often than once a month	101 (8.54)	54 (10.67)	0.166
Never	674 (57.02)	180 (35.57)	<0.001

Data presented as *n* (%).

a Chi-square tests used for analysis.

b Participants were asked to select all races and ethnicities with which they identified. Individuals selecting multiple identities were classified as multiracial.

### 3.2 Sample comparison results

**Figure 1:**
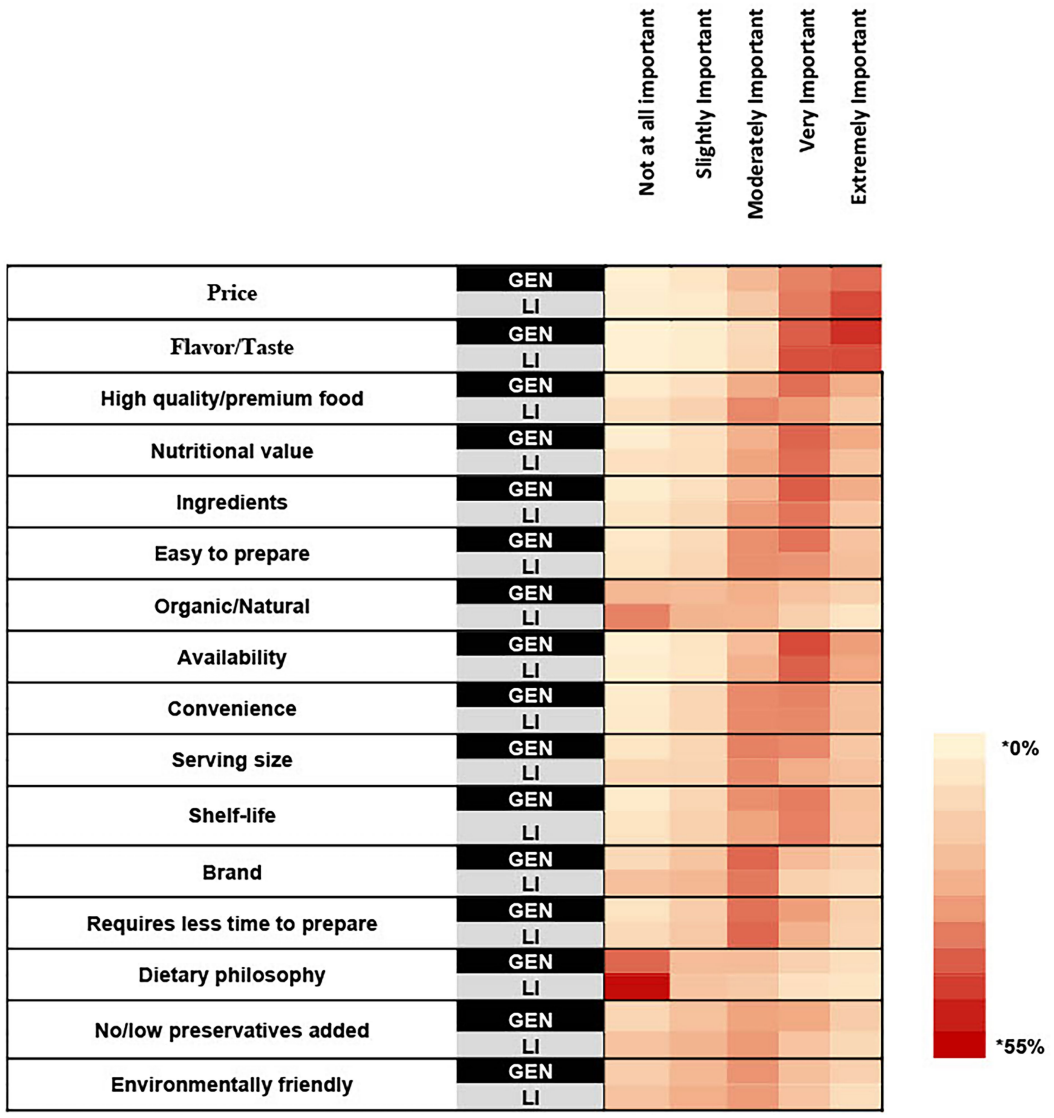
A heat map showing the percentage of respondents within each sample that selected each rating for how important the listed attributes are for household food purchasing. Survey question: How important are the following attributes when purchasing food for your household? * Cell color intensity represents the percentage of respondents within each sample who selected a given importance rating for each attribute. Darker shades indicate higher percentages.

**Figure 2:**
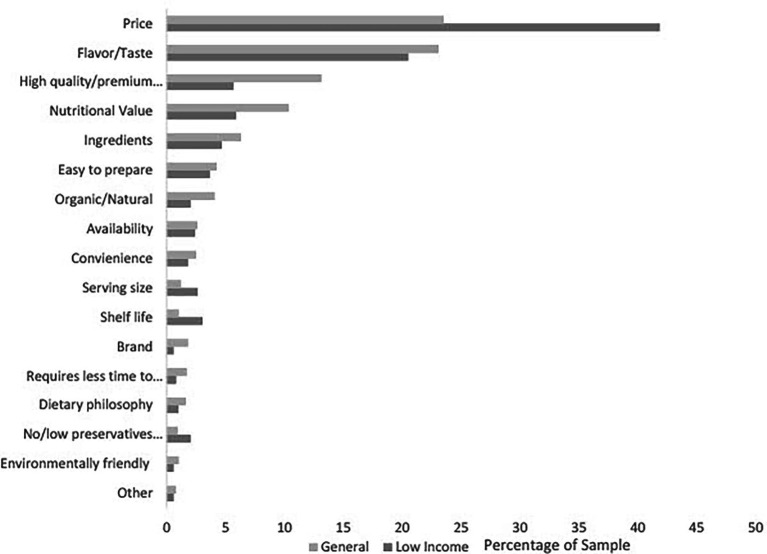
The percentage of respondents who selected each attribute as the most important for household food purchasing by sample.

Important attributes for food purchasing: When asked to rate the importance of food purchasing attributes, the attributes most frequently rated as “very” or “extremely important” for food purchasing in both samples were flavor/taste (GEN: 87.06%; LI: 85.18%), price (GEN: 71.32%; 78.26%), and availability (GEN: 72.25%; LI: 67.19%) (Figure 1). Compared to the general sample, a higher proportion of the low-income sample rated price as an important attribute for food purchasing (+6.94%; *p* = 0.003). There were no differences between the proportions of each sample that rated availability, or flavor/taste as “very” or “extremely important” for food purchasing. In both samples, across race and ethnicity, gender identity, and age groups, price, flavor/taste, and availability were rated as “very” or “extremely important” for food purchasing by the majority of each demographic (Supplementary Table 1). When asked to select the single most important attribute for food purchasing from the items previously rated as “very” or “extremely important,” the three most frequently selected attributes for food purchasing in the general sample were price (23.51%), flavor/taste (23.08%), and high-quality premium food (13.14%) (Figure 2). For the low-income sample, price (41.87%), flavor/taste (20.52%), and nutritional value (5.89%) were the three most frequently selected attributes (Figure 2).

### 3.3 Influential attributes for food purchasing

**Figure 3:**
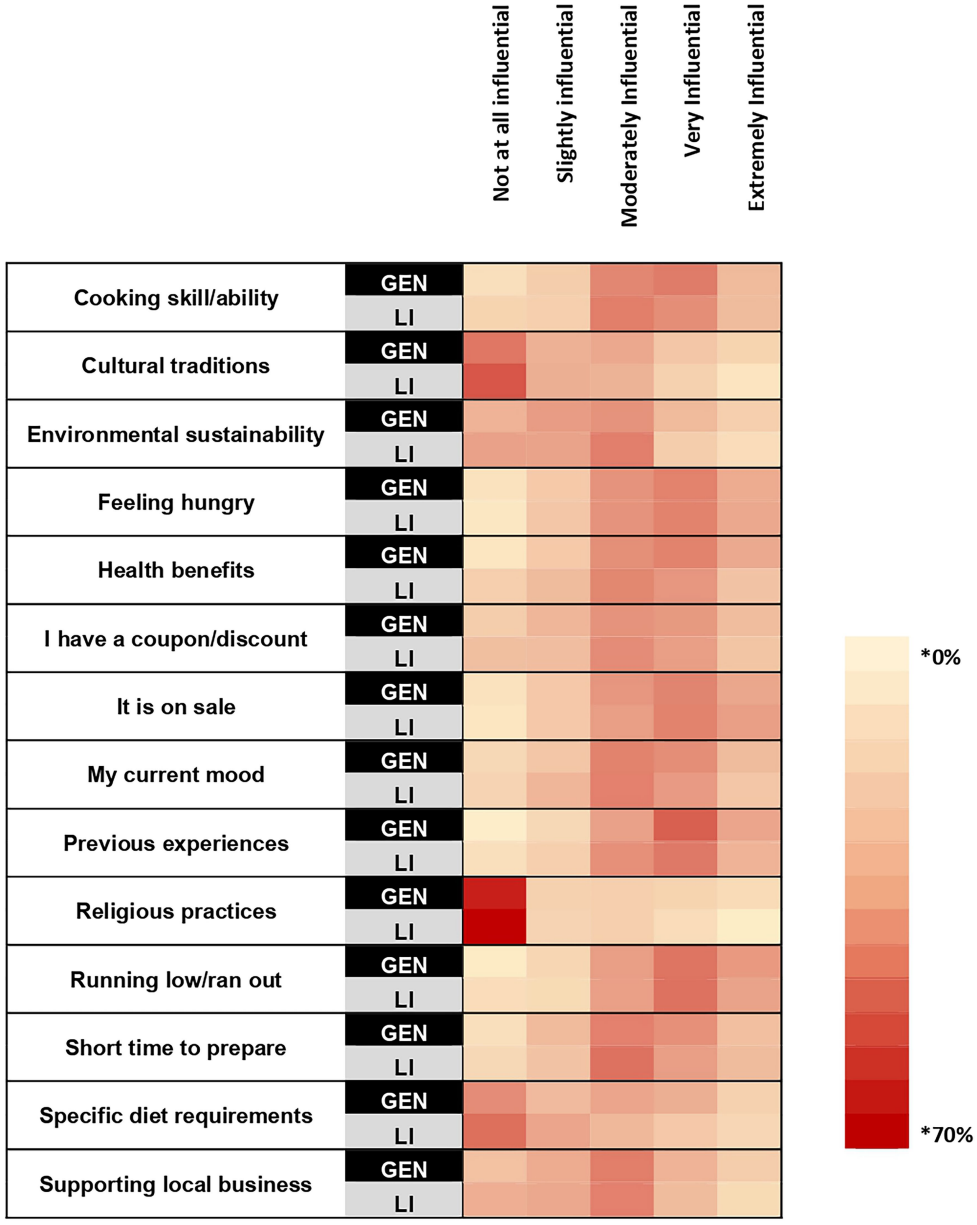
A heat map showing the percentage within each sample that selected each rating for how influential the listed attributes are for household food purchasing. Survey question: How influential are each of the following when purchasing food for your household? * Cell color intensity represents the percentage of respondents within each sample who selected a given importance rating for each attribute. Darker shades indicate higher percentages.

In the general sample, the top three most influential attributes for household food purchasing were “previous experience” (65.40%), “running low/ran out” (63.37%), and “it is on sale” (54.48%) (Figure 3). In the low-income sample, the top three most influential attributes for household food purchasing were “running low/ran out” (60.87%), “it is on sale” (57.71%), and “feeling hungry” (54.94%). A higher proportion of the general sample selected “previous experience,” than the low-income sample, as an influential attribute for household food purchasing (+11.65%; *p* < 0.001). There were no differences between the proportions of each sample selecting “running low/ran out,” “it is on sale,” and “feeling hungry” as influential attributes for household food purchasing.

In the general sample, across race and ethnicities, gender identities, and age groups, “previous experience” and “running low/ran out” of a food item were rated by the majority of each group as influential attributes for food purchasing. On the other hand, “it is on sale” was rated as influential by the majority of every demographic, but was not rated as influential by the majority of individuals identifying as American Indian or Alaska Native, Native Hawaiian or another Pacific Islander (Supplementary Table 2). In the low-income sample, the item “it is on sale” was rated as influential by the majority of every demographic group. The item “feeling hungry,” was rated as influential by the majority of every demographic except the 55 or older age group and those identifying as American Indian or Alaska Native, Native Hawaiian or another Pacific Islander. The item “running low/ran out” was rated as influential by the majority of every demographic group except for those identifying as Hispanic (Supplementary Table 2).

### 3.4 Attitudes about food purchasing

**Figure 4:**
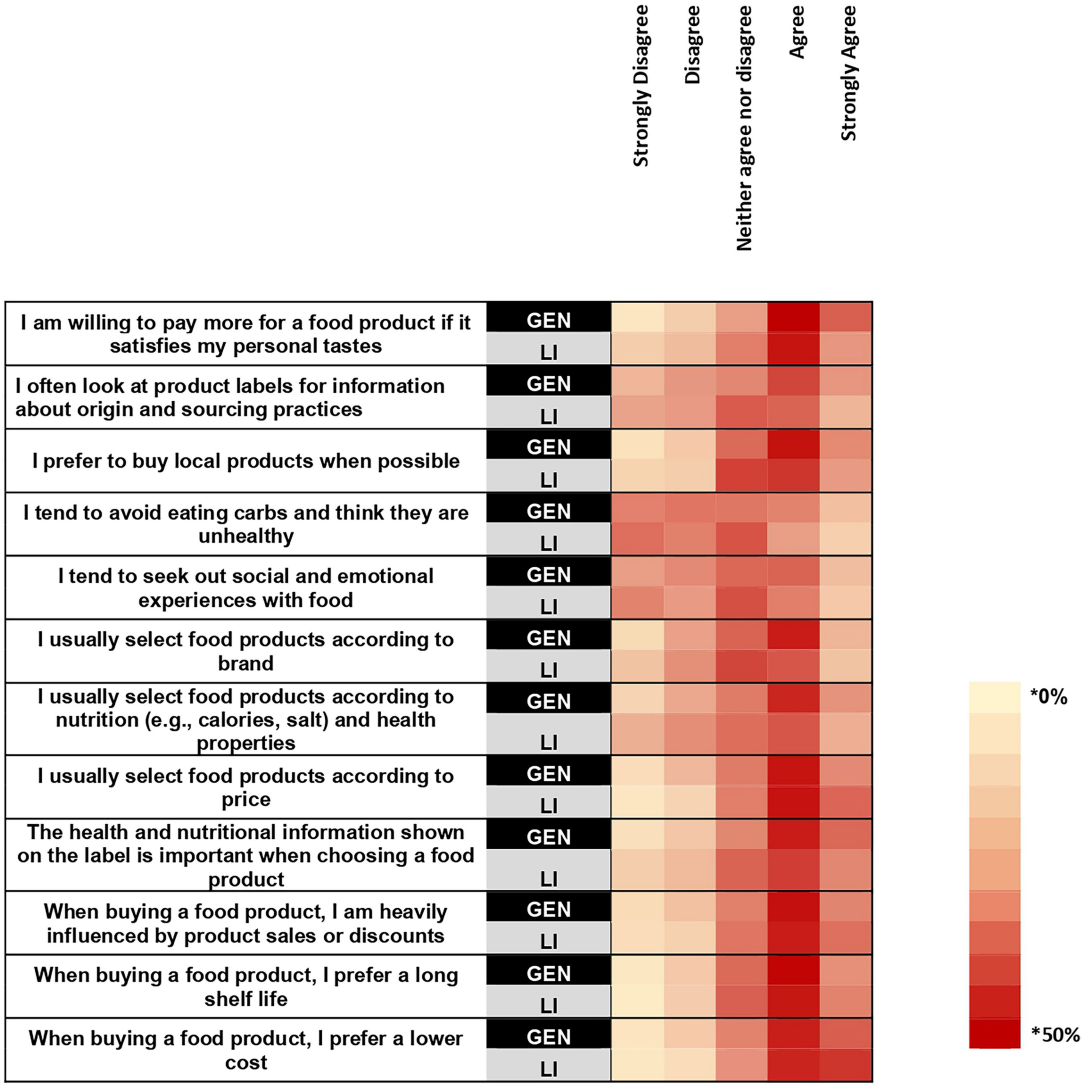
A heat map showing the percentage within each sample that selected each rating for how much they agree with the listed statements. Survey question: To what extent do you agree with each of the following statements? * Cell color intensity represents the percentage of respondents within each sample who selected a given importance rating for each attribute. Darker shades indicate higher percentages.

The three most frequently agreed with items in the general sample were “I am willing to pay more for a product if it satisfies my personal tastes” (73.35%), “when buying a food product, I prefer a lower cost” (67.85%), “the health and nutritional information shown on the label is important when choosing a food product” (66.67%) (Figure 4). The three most frequently agreed with items in the low-income sample were “when buying a food product, I prefer a lower cost” (74.31%), “I usually select foods according to price,” (68.58%), and “when buying a food product, I am heavily influenced by product sales or discounts” (65.02%). Higher proportions of the general sample, compared with the low-income sample, agreed with the statements “I am willing to pay more for a product if it satisfies my personal tastes” (+13.86%; *p* < 0.001) and “The health and nutrition information shown on the label is important when choosing a food product” (+11.93%; p < 0.001). A higher proportion of the low-income sample agreed with the statement “I usually select food products according to price” (+6.82%; *p* = 0.008). There was no difference between samples for “when buying a food product, I am heavily influenced by product sales or discounts.”

In the general sample, the items “I am willing to pay more for a product if it suits my personal tastes,” “The health and nutritional information shown on the label is important when choosing a food product,” and “The health and nutritional information shown on the label is important when choosing a food product” were agreed with by the majority of participants within each demographic group (Supplementary Table 3). In the low-income sample, the items “I usually select food products according to price” and “When buying a food product, I prefer a lower cost” were selected by the majority of participants in each demographic group (Supplementary Table 3). The item “When buying a food product, I am heavily influenced by product sales or discounts” was selected by the majority of every demographic group with the exception of those identifying as American Indian or Alaska Native, Native Hawaiian or Pacific Islander (Supplementary Table 3).

### 3.5 Barriers to healthy eating

In both the general sample and low-income samples, “price too high” (GEN: 53.13%; LI: 69.96%) and “does not taste as good” (GEN: 25.63%; LI: 24.11%) were the top two most frequently selected responses for barriers to healthy eating (Table 3). Reporting that “nothing prevents me from selecting healthier foods” was the third most frequently reported barrier for the general sample (24.37%), and “not as available” was the third for the low-income sample (16.60%). A higher proportion of the low-income sample, than the general sample reported price (+16.83%; *p* < 0.001) as barriers to choosing healthy foods whereas a higher proportion of the general sample reported nothing as a barrier (+9.15%; p < 0.001). There were no differences between samples in the proportion of each sample selecting “not as available.” Across most age groups, races and ethnicities, and gender identities in both samples, “price is too high” and “does not taste as good” were among the top three most frequently selected barriers (Supplementary Table 4). Within the general sample, the response option “nothing prevents me from selecting healthier foods” was not a top response in each demographic group, and there was variation across age, race and ethnicity, and gender identity groups for other commonly selected barriers.

**Table 3:** Frequency of selecting barriers and facilitators to healthy eating in each sample.

	General	Low-income
Facilitators		
Preparing grocery lists in advance	535 (45)	191 (38)
Good-tasting recipes	514 (43)	185 (37)
Budget-friendly recipes	413 (35)	181 (36)
Meal prepping in advance	335 (28)	97 (19)
Watching cooking videos	302 (26)	120 (24)
Access to the right cooking tools or equipment	183 (15)	57 (11)
Following health blogs/influencers	175 (15)	39 (8)
Recommendations from organizations	128 (11)	31 (6)
Consulting a dietician/nutrition	122 (10)	33 (7)
Nothing supports me in selecting healthier foods	184 (16)	127 (25)
Other	26 (2)	9 (2)
Barriers		
Price is too high	628 (53)	354 (70)
Does not taste as good	303 (26)	122 (24)
Shelf life too short	201 (17)	69 (14)
Preparation time is too long	192 (16)	57 (11)
Not as available	148 (13)	84 (17)
Serving size too small	158 (13)	49 (10)
I do not always have the right cooking tools or equipment	107 (9)	59 (12)
Nothing prevents me from selecting healthier foods	288 (24)	77 (15)
Other	21 (2)	8 (2)

Data presented as *n* (%).

The top three most frequently selected barriers to selecting whole grains in the general sample were taste (19.63%), cost (17.01%), and “I did not know this was recommended” (10.49%) (Table 4). In the low-income sample, the three most frequently selected barriers to choosing whole grains were cost (25.30%), taste (23.32%), and “I do not understand the difference” (13.04%). The top three most frequently selected barriers to selecting healthy proteins in the general sample were taste (19.37%), cost (17.60%), and “I do not care/try” (6.26%). In the low-income sample, the three most frequently selected barriers were cost (29.05%), taste (23.12%), “I do not understand the difference” (8.89%) and “I do not care” (8.89%). Higher proportions of the low-income sample selected cost as a barrier to selecting whole grains (+8.2%; *p* < 0.001) and proteins (+11.45%; p < 0.001), and not understanding which proteins are healthy choices as a barrier to choosing healthy proteins (+4.32%; p < 0.001). There were no differences between samples for selecting taste, not understanding the difference, and not knowing this was a recommendation as barriers to choosing whole grains. There were no differences for selecting taste or “I do not care/try” as barriers to choosing proteins. In both samples, across most age, race and ethnicity, and gender categories, cost and taste were among the top three most frequently selected barriers to selecting whole grains and healthy proteins (Supplementary Table 5). There was variation between demographic groups for other top barriers identified by each group in each sample.

**Table 4:** Frequency of and barriers to practicing healthy eating behaviors in each sample.

	General	Low income
How often do you choose whole grain over refined grain?		
Never	32 (3)	35 (7)
Rarely	145 (12)	72 (14)
Sometimes	476 (40)	234 (46)
Most of the time	380 (32)	130 (26)
Always	149 (13)	35 (7)
What prevents you from making at least half of your grains whole grains?		
Taste preferences	232 (20)	118 (23)
Cost	201 (17)	128 (25)
I do not understand the difference between whole and refined grains	118 (10)	66 (13)
I did not know this was recommended	124 (10)	55 (11)
I’m not sure who to use/prepare	90 (8)	39 (8)
Dietary restrictions	37 (3)	13 (3)
Cultural/religious preferences	16 (1)	6 (1)
I do not care/try	102 (9)	43 (8)
I do not know/unsure	59 (5)	39 (8)
Other	12 (1)	7 (1)
How often do you choose healthy instead of unhealthy sources of protein?		
Never	25 (2)	21 (4)
Rarely	77 (7)	65 (13)
Sometimes	419 (35)	213 (42)
Most of the time	504 (43)	159 (31)
Always	157 (13)	48 (9)
What prevents you from choosing healthy sources of protein?		
Cost	208 (18)	147 (29)
Taste preferences	229 (19)	117 (23)
I did not know this was recommended	55 (5)	20 (4)
I’m not sure how to use/prepare	40 (3)	24 (5)
Dietary restrictions	28 (2)	10 (2)
Cultural/religious preferences	13 (1)	5 (1)
I do not understand which are healthy choices	54 (5)	45 (9)
I do not care/try	74 (6)	45 (9)
I do not know/unsure	33 (3)	28 (6)
Other	19 (2)	5 (1)
None of the above	21 (2)	11 (2)

Data presented as *n* (%).

### 3.6 Facilitators to healthy eating

The top three selected facilitators for healthy eating in both samples were “preparing grocery lists in advance” (GEN 45.26%; LI 37.75%), “good tasting recipes” (GEN 43.49%; LI 36.56%), and “budget friendly recipes” (GEN 34.94%; LI 35.77%) (Table 3). A higher proportion of the general sample selected “preparing lists in advance” (+7.51%; *p* = 0.004) and “good tasting recipes” (+6.93%; *p* = 0.008) as supports and there were no differences between samples in the proportion selecting “budget friendly recipes” as a support. For facilitators to healthy eating, in the general sample, “preparing grocery lists in advance” was a top response in all demographic groups except for individuals identifying as multi-racial. The response “good tasting recipes,” was a top response in every demographic group except those identifying as non-binary, and “budget friendly recipes” was a top response for all demographic groups except for those identifying as multi-racial or those identifying as American Indian or Alaska Native, Native Hawaiian or Pacific Islander (Supplementary Table 4). In the low-income sample, “preparing grocery lists in advance” was a top response among most demographics, except the 18–24 age group, and those identifying as Hispanic, Asian, or Men. The response “Good tasting recipes” was a top response for all demographics except those identifying as American Indian or Alaska Native, Native Hawaiian or Pacific Islanders, and the response “budget-friendly recipes was a support among all demographics except for individuals identifying as Black.

## 4 Discussion

This study aimed to explore differences in barriers and facilitators to healthy eating between a US census-representative sample of the general population and a sample of low-income individuals receiving federal benefits, and to explore barriers and facilitators to healthy eating between demographic groups within each sample. Taste and cost constraints were consistently identified as barriers to choosing healthy foods in both the general and low-income samples and between all demographic groups within each sample. Larger proportions of participants in the low-income sample than the general sample reported cost as a barrier to healthy eating whereas there were no differences between the samples for reporting taste as a barrier to healthy eating. The availability of healthy foods was also found to be a barrier to healthy eating in both samples. Furthermore, while the majority of both samples agreed that the health and nutritional information on food labels is important, the overall percentage of each sample selecting nutritional value as the most important attribute for food purchasing was small. In addition, it was found that preparing grocery lists in advance and having access to good tasting and budget friendly recipes were supports to healthy eating in both samples. Taken together, these findings suggest food purchasing priorities for consumers are centered on the cost and taste of foods, are not centered on nutritional value, and may be influenced by healthy food availability.

The findings that cost and taste are top food purchasing priorities and barriers (real or perceived) to healthy eating are consistent with other studies in a wide variety of populations (17, 24–27). Furthermore, in the yearly Food and Health Survey completed by the International Food Information Council (IFIC) that assesses consumer attitudes toward food, nutrition and health, taste and food price have been ranked as the top two most important drivers for food purchasing decisions since 2006 (28). Additionally, it is also important to note that cost and taste priorities have been consistently rated as more important than priorities such as healthfulness, convenience, and environmental sustainability (28). In the present study, although cost was identified as a barrier to healthy eating in both samples and across all demographic groups, it was selected by a larger proportion of the low-income sample. This finding is consistent with other studies that suggest cost is a barrier of critical significance for those with a low-income receiving federal benefits, such as SNAP (29). Furthermore, the importance of cost as a determinate of food choice can also be observed when considering diet quality or nutrition security trends by socioeconomic status. Specifically, although diet quality is low across all socioeconomic gradients in the U.S., it is considerably worse in lower socioeconomic status households relative to higher socioeconomic status households (30).

In addition to cost, the taste and availability of healthy foods were also identified as key barriers to healthy eating. A large body of evidence suggests that taste preference is a key determinate of food choice (31–33). Although there are a wide variety of tasty healthy food options, healthy foods are thought to be “less tasty” than unhealthy foods (34), which may reduce consumption of healthy options, such as fruits and vegetables (35), whole grains (36), and lean proteins (37). Thus, it is unsurprising that taste was identified as a barrier to choosing healthy foods across samples, age groups, race and ethnicities, and gender identities. Perceptions that unhealthy foods are tasty, and healthy foods are not, may be directly related to the ubiquitous presence of foods that are both unhealthy and tasty in the food environment (38). Thus, the proportional availability of unhealthy relative to healthy foods in the current food environment is likely a driver of taste preferences and thus food choice. Indeed, perceptions of the taste-health relationship can be altered by more frequent exposures to healthy tasty foods (38).

Beyond cost, taste, and availability, there are other barriers that may make consuming a healthy diet difficult or unachievable in low-income populations receiving federal benefits. One such example is the time constraints associated with meal preparation (26, 29, 39, 40). While previous literature has identified time constraints associated with food preparation as a barrier to healthy eating (17, 41), this was not a barrier identified by the present study. Specifically, items related to time constraints for meal preparation, such as “short preparation time,” or “convenience,” were not found to be top ranked attributes of influence for household food purchasing in either sample or across demographic categories. One potential explanation for the discrepancy in these findings is that this survey was not validated. Thus, the items designed to capture the importance of time constraints in meal preparation may not reflect participants’ true beliefs or attitudes about the influences of this barrier on healthy food choices.

In addition to identifying barriers to healthy eating, the present study also identified facilitators to selecting healthy foods. Specifically, having access to good tasting and budget-friendly recipes in addition to planning grocery lists in advance were identified as supports to choosing healthy foods in both samples. In other populations, supports to healthy eating often center on increased availability of healthy food options (42–45). Although (lack of) availability was assessed as a barrier to choosing healthy foods, increased availability was not a construct assessed as a facilitator to choosing healthy foods in the present study. While recipes for tasty and budget-friendly foods are not increasing the physical presence of food items in communities, providing examples of meals that are tasty, healthy, and budget-friendly may increase perceived accessibility of healthy eating through promoting self-efficacy. In a recent systematic review investigating the effects of culinary education interventions on dietary intake and behavior it was found that culinary focused interventions increased self-efficacy and attitudes around healthy eating, which translated to improved diet quality (46). Taken together, more research is needed to confirm that providing good tasting and budget friendly recipes increases the perceived accessibility of healthy eating and thus serves to facilitate healthy eating and improved diet quality.

Collectively, the findings of this study add to the literature by confirming and quantifying taste, cost and availability as barriers to healthy eating. As such, focusing interventions on addressing concerns regarding taste, cost and availability may be promising directions for future studies seeking to improve diet quality. Specifically, emphasizing the taste of healthy foods rather than their health benefits, and focusing on reducing the costs and increasing the availability of healthy foods may be beneficial intervention targets to improve diet quality. In alignment, an intervention designed to increase vegetable intake in a food buffet found that taste-focused labels increased vegetable intake by 29 and 14% when compared with health-focused or basic labels, respectively (47). Regarding cost and availability as barriers to healthy diets, it is currently not clear how to best address these barriers from an intervention perspective. While monetary food assistance programs are known to improve food security, evidence is mixed for their effects on diet quality (48), which may be in part because the food environment is characterized by an over-abundance of energy-dense, nutrient-poor foods (49). In contrast, limited evidence suggests diet quality may be modestly increased through providing subsidized subscriptions to community supported agriculture programs (50), or through providing fruit and vegetable vouchers (51) or prescriptions (52). Taken together, more research is needed to best understand how to effectively address the cost and availability of healthy foods as barriers to healthy eating within the context of the current food environment.

The present study must be considered in light of its strengths and limitations. In terms of its strengths, this study had a large sample size that allowed for quantitative comparisons between a general and a low-income sample of US adults. This is a strength because through providing quantitative comparisons between these populations, this study offers novel contributions to an evidence base largely comprised of qualitative findings. Specifically, this study highlights that cost and taste are critical barriers to healthy eating across multiple levels of income, and across multiple demographic groups in the US. Thus, quantifying the proportion of individuals experiencing cost and taste as barriers to healthy eating within these demographics highlights the utility of cost and taste as critical intervention targets for improving population-level diet quality.

This study has several limitations. First, the survey instrument used in this study has not been validated for the constructs assessed, and it is unclear if the questions were received as intended. In addition, there may have been constructs, such as time constraints and availability, that were not adequately assessed with this instrument. However, the strong emergence of cost and taste as barriers to healthy eating across all survey items and the concordance between these findings and the larger body of literature in this area indicate that the constructs of taste and cost were adequately assessed by this survey instrument. Second, while the general sample was census representative for some characteristics, both the general and low-income samples were predominately White, which limits the generalizability of these results to other racial and ethnic groups. Next, the criteria used to define the low-income population may not have adequately captured individuals experiencing poverty, because census poverty thresholds are determined by income in addition to family size. Thus, selecting one income threshold for all individuals regardless of family size is a limitation. This limitation was mitigated through the additional requirement of receiving federal benefits to be classified as low-income in the present study. Lastly, it is a limitation that frequency of skipping a meal was used as a proxy for food insecurity in place of using a validated food insecurity assessment tool.

In conclusion, taste and cost were frequently reported barriers to choosing healthy foods in both a general and a low-income sample of U.S. adults, although cost was a barrier to a larger proportion of the low-income sample than the general sample. Future interventions focusing on improving diet quality may benefit from considering cost and taste as critical barriers to healthy eating.

## Data Availability

The original data will be shared upon reasonable request to the corresponding author.
