# Supplementary material for: Household barriers and facilitators to healthy eating in a U.S. census-representative sample of the general population and a low-income sample: a cross-sectional survey
Source: Front Public Health. 2025 Aug 13;13:1648218. doi: 10.3389/fpubh.2025.1648218 (PMC12381678; doi:10.3389/fpubh.2025.1648218)
Supplement: Supplementary file 1 [file Data_Sheet_1.docx]

# Supplementary Material

## ***Supplementary Table 1: The frequency and percentage of respondents selecting “very” or “extremely” important for each food purchasing attribute.***

|  |  | Age Group (years) | | | | | Race and Ethnicity | | | | | | Gender | | |
| --- | --- | --- | --- | --- | --- | --- | --- | --- | --- | --- | --- | --- | --- | --- | --- |
|  | Sample | 18-24 | 25-34 | 35-44 | 45-54 | 55+ | White | Black | Hispanic | Asian | Multiracial | Other | Male | Female | Non-binary |
| Price | GEN | 104(74) | 147(77) | 172(74) | 126(68) | 294(68) | 605(71) | 104(75) | 40(65) | 45(76) | 12(67) | 37(73) | 399(68) | 443(74) | 1(50) |
|  | LI | 52(72) | 49(77) | 60(75) | 77(84) | 158(80) | 295(80) | 44(71) | 11(69) | 8(89) | 7(100) | 31(82) | 142(77) | 250(79) | 4(67) |
| Flavor/taste | GEN | 107(76) | 155(81) | 199(85) | 169(92) | 399(92) | 764(89) | 104(75) | 51(82) | 51(86) | 15(83) | 44(86) | 492(84) | 535(90) | 2(100) |
|  | LI | 60(83) | 53(83) | 67(84) | 77(84) | 174(88) | 315(86) | 54(77) | 14(88) | 8(89) | 7(100) | 33(87) | 155(84) | 270(85) | 6(100) |
| High quality/premium food | GEN | 70(50) | 126(66) | 153(66) | 113(61) | 285(66) | 531(62) | 102(74) | 35(56) | 41(69) | 9(50) | 29(57) | 378(65) | 368(62) | 1(50) |
|  | LI | 21(29) | 25(39) | 39(49) | 46(50) | 103(52) | 169(46) | 37(36) | 7(44) | 5(56) | 2(29) | 14(37) | 83(45) | 149(47) | 2(33) |
| Nutritional value | GEN | 83(59) | 126(66) | 161(69) | 117(64) | 290(67) | 561(66) | 100(72) | 36(58) | 37(63) | 13(72) | 30(59) | 367(63) | 409(69) | 1(50) |
|  | LI | 37(51) | 36(56) | 46(58) | 50(54) | 116(59) | 200(54) | 40(52) | 9(56) | 9(100) | 4(57) | 23(61) | 103(56) | 178(56) | 4(67) |
| Ingredients | GEN | 92(65) | 127(66) | 157(67) | 124(67) | 281(65) | 541(63) | 113(82) | 36(58) | 46(78) | 11(61) | 34(67) | 380(65) | 399(67) | 2(100) |
|  | LI | 35(49) | 35(55) | 46(58) | 49(53) | 107(54) | 188(51) | 47(51) | 9(56) | 3(33) | 5(71) | 20(53) | 92(50) | 177(56) | 3(50) |
| Easy to prepare | GEN | 84(60) | 112(58) | 138(59) | 95(52) | 211(49) | 452(53) | 82(59) | 34(55) | 34(58) | 11(61) | 27(53) | 310(53) | 330(55) | 0(0) |
|  | LI | 35(49) | 34(53) | 46(58) | 49(53) | 94(47) | 187(51) | 40(49) | 7(44) | 4(44) | 3(43) | 17(45) | 94(51) | 162(51) | 2(33) |
| Organic/Natural | GEN | 57(40) | 80(42) | 95(41) | 58(32) | 91(21) | 231(27) | 75(54) | 17(27) | 27(46) | 10(56) | 21(41) | 210(36) | 171(29) | 0(0) |
|  | LI | 23(32) | 11(17) | 23(29) | 15(16) | 25(13) | 51(14) | 25(16) | 5(31) | 4(44) | 3(43) | 9(24) | 43(23) | 54(17) | 0(0) |
| Availability | GEN | 86(61) | 135(70) | 172(74) | 147(80) | 314(73) | 619(72) | 102(74) | 40(65) | 42(71) | 14(78) | 37(73) | 405(69) | 447(75) | 2(100) |
|  | LI | 43(60) | 44(69) | 55(69) | 69(75) | 129(65) | 244(66) | 52(64) | 8(50) | 6(67) | 4(57) | 26(68) | 125(68) | 211(67) | 4(67) |
| Convenience | GEN | 82(58) | 112(58) | 146(63) | 100(54) | 191(44) | 441(52) | 86(62) | 33(53) | 38(64) | 9(50) | 24(47) | 315(54) | 316(53) | 0(0) |
|  | LI | 40(56) | 29(45) | 47(59) | 60(65) | 91(46) | 189(51) | 44(42) | 5(31) | 6(67) | 2(29) | 21(55) | 100(54) | 164(52) | 3(50) |
| Serving size | GEN | 80(57) | 104(54) | 130(56) | 91(49) | 174(40) | 386(45) | 92(67) | 29(47) | 35(59) | 9(50) | 28(55) | 299(51) | 280(47) | 0(0) |
|  | LI | 33(46) | 29(45) | 44(55) | 38(41) | 83(42) | 151(41) | 41(42) | 8(50) | 5(56) | 4(57) | 18(47) | 85(46) | 141(45) | 1(17) |
| Shelf-life | GEN | 82(58) | 120(63) | 125(54) | 91(49) | 216(50) | 431(50) | 97(70) | 33(53) | 35(59) | 9(50) | 29(57) | 317(54) | 315(53) | 2(100) |
|  | LI | 28(39) | 31(48) | 43(54) | 51(55) | 111(56) | 184(50) | 40(45) | 10(63) | 7(78) | 4(57) | 19(50) | 92(50) | 169(53) | 3(50) |
| Brand | GEN | 58(41) | 77(40) | 80(34) | 55(30) | 129(30) | 265(31) | 82(59) | 14(23) | 16(27) | 7(39) | 15(29) | 233(40) | 166(28) | 0(0) |
|  | LI | 18(25) | 20(31) | 23(29) | 23(25) | 34(17) | 72(20) | 28(29) | 7(44) | 2(22) | 2(29) | 7(18) | 54(29) | 64(20) | 0(0) |
| Requires less time to prepare | GEN | 57(40) | 96(50) | 111(48) | 78(42) | 160(37) | 361(42) | 69(50) | 19(31) | 25(42) | 8(44) | 20(39) | 241(41) | 261(44) | 0(0) |
|  | LI | 28(39) | 25(39) | 33(41) | 38(41) | 58(29) | 127(35) | 31(36) | 5(31) | 3(33) | 1(14) | 15(39) | 65(35) | 114(36) | 3(50) |
| Dietary Philosophy | GEN | 37(26) | 60(31) | 59(25) | 31(17) | 58(13) | 164(19) | 49(36) | 9(15) | 9(15) | 4(22) | 10(20) | 132(23) | 113(19) | 0(0) |
|  | LI | 12(17) | 7(11) | 15(19) | 8(9) | 22(11) | 38(10) | 15(10) | 3(19) | 0(0) | 2(29) | 6(16) | 36(20) | 28(9) | 0(0) |
| No/low preservatives added | GEN | 56(40) | 95(49) | 103(44) | 79(43) | 160(37) | 334(39) | 77(56) | 27(44) | 27(46) | 8(44) | 20(39) | 235(40) | 258(43) | 0(0) |
|  | LI | 16(22) | 13(20) | 24(30) | 23(25) | 65(33) | 95(26) | 24(19) | 6(38) | 4(44) | 2(29) | 10(26) | 50(27) | 91(29) | 0(0) |
| Environmentally friendly | GEN | 55(39) | 87(45) | 85(36) | 48(26) | 110(25) | 253(30) | 73(53) | 12(19) | 19(32) | 6(33) | 22(43) | 198(34) | 187(31) | 0(0) |
|  | LI | 19(26) | 17(27) | 23(29) | 27(29) | 47(24) | 87(24) | 26(25) | 6(38) | 2(22) | 3(43) | 9(24) | 57(31) | 75(24) | 1(17) |

Data presented as n(%)

## ***Supplementary Table 2: The frequency and percentage of respondents selecting “very” or “extremely” influential for each food purchasing attribute.***

|  | Age group (years) | |  |  |  | Race and Ethnicity | |  |  |  |  | Gender Identity | |  |
| --- | --- | --- | --- | --- | --- | --- | --- | --- | --- | --- | --- | --- | --- | --- |
| Item | 18-24 | 25-34 | 35-44 | 44-55 | 55+ | White | Black | Hispanic | Asian | Other | Multiracial | Male | Female | Non-Binary |
| Cooking skill/Ability | | |  |  |  |  |  |  |  |  |  |  |  |  |
| GEN | 77(43) | 112(31) | 130(26) | 96(21) | 186(12) | 421(49) | 83(60) | 34(55) | 32(54) | 31(50) | 27(53) | 302(53) | 298(50) | 1(50) |
| LI | 34(15) | 33(20) | 45(31) | 47(11) | 70(9) | 156(43) | 38(55) | 12(75) | 3(33) | 3(50) | 17(45) | 79(43) | 149(47) | 1(20) |
| Cultural Traditions | |  |  |  |  |  |  |  |  |  |  |  |  |  |
| GEN | 59(43) | 60(31) | 60(26) | 38(21) | 53(12) | 150(18) | 70(51) | 14(23) | 19(32) | 3(38) | 14(27) | 160(28) | 109(18) | 1(50) |
| LI | 11(15) | 13(20) | 25(31) | 10(11) | 18(9) | 46(13) | 18(26) | 4(25) | 3(33) | 2(33) | 4(11) | 36(20) | 41(13) | 0(0) |
| Environmental Sustainability | | |  |  |  |  |  |  |  |  |  |  |  |  |
| GEN | 54(40) | 74(39) | 72(31) | 46(25) | 77(18) | 197(23) | 73(53) | 13(21) | 17(29) | 4(50) | 19(37) | 172(30) | 150(25) | 1(50) |
| LI | 16(22) | 11(17) | 22(28) | 19(21) | 26(13) | 55(15) | 24(35) | 4(25) | 0(0) | 2(33) | 9(24) | 47(26) | 47(15) | 0(0) |
| Feeling hungry | |  |  |  |  |  |  |  |  |  |  |  |  |  |
| GEN | 90(65) | 120(63) | 140(61) | 104(57) | 180(42) | 443(52) | 92(67) | 34(55) | 32(54) | 4(50) | 29(57) | 318(55) | 315(53) | 1(50) |
| LI | 45(63) | 40(63) | 55(69) | 51(55) | 87(44) | 194(53) | 40(58) | 9(56) | 7(78) | 2(33) | 26(68) | 103(56) | 172(54) | 3(60) |
| Health benefits | |  |  |  |  |  |  |  |  |  |  |  |  |  |
| GEN | 77(57) | 114(59) | 130(57) | 95(52) | 216(50) | 438(51) | 90(65) | 34(55) | 37(63) | 5(63) | 28(55) | 306(53) | 325(55) | 1(50) |
| LI | 25(35) | 30(47) | 40(50) | 31(34) | 83(42) | 140(38) | 33(48) | 8(50) | 5(56) | 3(50) | 20(53) | 73(40) | 133(42) | 3(60) |
| I have a coupon/discount | | |  |  |  |  |  |  |  |  |  |  |  |  |
| GEN | 74(53) | 85(44) | 111(48) | 77(42) | 148(34) | 347(41) | 72(52) | 21(34) | 30(51) | 3(38) | 22(43) | 242(42) | 252(42) | 1(50) |
| LI | 28(39) | 29(45) | 31(39) | 38(41) | 68(35) | 133(36) | 29(42) | 7(44) | 5(56) | 4(67) | 16(42) | 64(35) | 127(40) | 3(60) |
| It is on sale | |  |  |  |  |  |  |  |  |  |  |  |  |  |
| GEN | 77(56) | 114(59) | 123(53) | 107(59) | 217(51) | 463(54) | 76(55) | 31(50) | 37(63) | 3(38) | 28(55) | 295(51) | 342(58) | 1(50) |
| LI | 37(51) | 37(58) | 48(60) | 63(68) | 106(54) | 215(59) | 38(55) | 8(50) | 6(67) | 5(83) | 19(50) | 100(55) | 188(59) | 3(60) |
| My current mood | |  |  |  |  |  |  |  |  |  |  |  |  |  |
| GEN | 80(57) | 111(58) | 121(52) | 93(51) | 133(31) | 363(43) | 81(59) | 34(55) | 25(42) | 3(38) | 32(63) | 251(43) | 286(48) | 1(50) |
| LI | 33(46) | 29(45) | 43(54) | 40(43) | 53(27) | 138(38) | 30(43) | 6(38) | 4(44) | 3(50) | 17(45) | 67(36) | 128(41) | 3(60) |
| Previous experience | | |  |  |  |  |  |  |  |  |  |  |  |  |
| GEN | 80(59) | 117(61) | 158(69) | 121(66) | 291(68) | 560(66) | 94(68) | 35(56) | 39(66) | 4(50) | 35(69) | 386(67) | 379(64) | 2(100) |
| LI | 36(50) | 35(55) | 43(54) | 49(53) | 108(55) | 196(53) | 33(48) | 7(44) | 5(56) | 4(67) | 26(68) | 102(56) | 168(53) | 1(20) |
| Religious practices | |  |  |  |  |  |  |  |  |  |  |  |  |  |
| GEN | 42(30) | 53(28) | 59(25) | 20(11) | 32(7) | 115(13) | 62(45) | 4(6) | 14(24) | 2(25) | 9(18) | 129(22) | 77(13) | 0(0) |
| LI | 10(14) | 5(8) | 18(23) | 6(7) | 10(5) | 24(7) | 19(28) | 1(6) | 1(11) | 2(33) | 2(5) | 29(6) | 20(0) | 0(0) |
| Running low/ran out | | |  |  |  |  |  |  |  |  |  |  |  |  |
| GEN | 75(55) | 127(66) | 151(65) | 117(64) | 274(64) | 543(64) | 90(65) | 32(52) | 38(64) | 6(75) | 35(69) | 338(59) | 404(68) | 2(100) |
| LI | 40(56) | 38(59) | 51(64) | 60(65) | 119(60) | 232(63) | 37(54) | 6(38) | 5(56) | 3(50) | 25(66) | 110(60) | 195(62) | 3(60) |
| Short time to prepare | | |  |  |  |  |  |  |  |  |  |  |  |  |
| GEN | 62(45) | 100(52) | 124(54) | 81(44) | 147(34) | 364(43) | 69(50) | 26(42) | 25(42) | 3(38) | 27(53) | 242(42) | 271(46) | 1(50) |
| LI | 31(43) | 26(41) | 36(45) | 41(45) | 72(36) | 150(41) | 32(46) | 4(25) | 4(44) | 2(33) | 14(37) | 75(41) | 128(41) | 3(60) |
| Specific diet requirements | | |  |  |  |  |  |  |  |  |  |  |  |  |
| GEN | 50(36) | 74(39) | 89(39) | 48(26) | 95(22) | 238(28) | 66(48) | 15(24) | 18(31) | 4(50) | 15(29) | 177(31) | 177(30) | 2(100) |
| LI | 20(28) | 14(22) | 22(28) | 18(20) | 35(18) | 69(19) | 21(30) | 3(19) | 2(22) | 3(50) | 11(29) | 49(27) | 59(19) | 1(20) |
| Supporting local business |  |  |  |  |  |  |  |  |  |  |  |  |  |  |
| GEN | 53(38) | 76(40) | 74(32) | 51(28) | 103(24) | 248(29) | 67(49) | 13(21) | 15(25) | 1(13) | 13(25) | 191(33) | 165(28) | 1(50) |
| LI | 17(28) | 11(22) | 24(28) | 27(20) | 43(18) | 84(19) | 21(30) | 6(19) | 2(22) | 2(50) | 7(29) | 52(27) | 69(19) | 1(20) |

Data presented as n(%)

## ***Supplementary Table 3: The frequency and percentage of respondents selecting “agree” or “strongly agree” for each food purchasing attitude.***

|  | **Age group (years)** | | | | | **Race** | | | |  |  | **Gender** |  |  |
| --- | --- | --- | --- | --- | --- | --- | --- | --- | --- | --- | --- | --- | --- | --- |
| **Item by sample** | **18-24** | **25-34** | **35-44** | **44-55** | **55+** | **White** | **Black** | **Hispanic** | **Asian** | **Multiple** | **Other** | **Male** | **Female** | **Non-binary** |
| **I am willing to pay more for a food product if it satisfies my personal tastes** |  |  |  |  |  |  |  |  |  |  |  |  |  |  |
| GEN | 101(72)* | 145(76)* | 169(73) | 131(71) | 321(74) | 634(74) | 97(70) | 46(74) | 40(68) | 4(50) | 38(75) | 427(73) | 440(74) | 0(0) |
| LI | 48(67) | 35(55) | 53(66) | 52(57) | 113(57) | 222(60) | 39(57) | 10(63) | 4(44) | 5(83) | 21(55) | 112(61) | 185(59) | 4(67) |
| **I often look at product labels for information about origin and sourcing practices** |  |  |  |  |  |  |  |  |  |  |  |  |  |  |
| GEN | 82(58) | 95(49) | 127(55) | 91(49) | 200(46) | 258(46) | 51(68) | 21(52) | 30(66) | 3(75) | 19(49) | 184(51) | 200(49) | 1(50) |
| LI | 32(44) | 25(39) | 34(43) | 28(30) | 79(40) | 138(38) | 31(45) | 11(69) | 4(44) | 4(67) | 10(26) | 69(38) | 127(40) | 2(33) |
| **I prefer to buy local products when possible** |  |  |  |  |  |  |  |  |  |  |  |  |  |  |
| GEN | 78(55) | 127(66) | 143(61) | 110(60) | 279(65) | 535(63) | 92(67) | 30(48) | 37(63) | 4(50) | 34(67) | 357(61) | 378(63) | 2(100) |
| LI | 27(38) | 32(50) | 34(43) | 50(54) | 123(62) | 206(56) | 29(42) | 7(44) | 2(22) | 4(67) | 17(45) | 91(49) | 170(54) | 5(83) |
| **I tend to avoid eating carbs and think they are unhealthy** |  |  |  |  |  |  |  |  |  |  |  |  |  |  |
| GEN | 51(36) | 68(35) | 83(36) | 59(32) | 108(25) | 241(28) | 63(46) | 23(37) | 24(41) | 3(38) | 13(25) | 190(33) | 179(30) | 0(0) |
| LI | 18(25) | 15(23) | 21(26) | 19(21) | 43(22) | 69(19) | 23(33) | 4(25) | 3(33) | 4(67) | 13(34) | 44(24) | 71(22) | 1(17) |
| **I tend to seek out social and emotional experiences with food** |  |  |  |  |  |  |  |  |  |  |  |  |  |  |
| GEN | 74(52) | 97(51) | 109(47) | 65(35) | 96(22) | 298(35) | 63(46) | 24(39) | 24(41) | 3(38) | 26(51) | 221(38) | 219(37) | 1(50) |
| LI | 30(42) | 24(38) | 33(41) | 26(28) | 43(22) | 109(30) | 23(33) | 5(31) | 5(56) | 4(67) | 10(26) | 54(29) | 98(31) | 4(67) |
| **I usually select food products according to brand** |  |  |  |  |  |  |  |  |  |  |  |  |  |  |
| GEN | 22(51) | 33(62) | 29(56) | 17(47) | 40(49) | 86(51) | 38(63) | 9(56) | 4(46) | 1(50) | 2(49) | 75(54) | 66(51) | .(50) |
| LI | 23(32) | 27(42) | 32(40) | 36(39) | 79(40) | 136(37) | 37(54) | 5(31) | 4(44) | 4(67) | 11(29) | 79(43) | 117(37) | 1(17) |
| **I usually select food products according to nutrition (e.g., calories, salt) and health properties** |  |  |  |  |  |  |  |  |  |  |  |  |  |  |
| GEN | 82(58) | 108(56) | 140(60) | 102(55) | 239(55) | 472(55) | 86(62) | 38(61) | 33(56) | 6(75) | 31(61) | 317(54) | 354(59) | 0(0) |
| LI | 31(43) | 26(41) | 33(41) | 37(40) | 89(45) | 149(41) | 31(45) | 10(63) | 6(67) | 5(83) | 15(39) | 78(42) | 136(43) | 2(33) |
| **I usually select food products according to price** |  |  |  |  |  |  |  |  |  |  |  |  |  |  |
| GEN | 94(67) | 129(67) | 157(67) | 116(63) | 234(54) | 523(61) | 87(63) | 36(58) | 38(64) | 4(50) | 34(67) | 333(57) | 395(66) | 2(100) |
| LI | 49(68) | 47(73) | 60(75) | 59(64) | 132(67) | 258(70) | 44(64) | 8(50) | 7(78) | 6(100) | 23(61) | 125(68) | 219(69) | 4(67) |
| **The health and nutritional information shown on the label is important when choosing a food product** |  |  |  |  |  |  |  |  |  |  |  |  |  |  |
| GEN | 89(63) | 131(68) | 164(70) | 117(64) | 283(66) | 554(65) | 95(69) | 48(77) | 46(78) | 6(75) | 35(69) | 376(64) | 410(69) | 2(100) |
| LI | 42(58) | 30(47) | 39(49) | 45(49) | 121(61) | 190(52) | 40(58) | 11(69) | 6(67) | 5(83) | 24(63) | 109(59) | 164(52) | 4(67) |
| **When buying a food product, I am heavily influenced by product sales or discounts** |  |  |  |  |  |  |  |  |  |  |  |  |  |  |
| GEN | 89(63) | 123(64) | 149(64) | 124(67) | 265(61) | 546(64) | 84(61) | 38(61) | 38(64) | 5(63) | 33(65) | 354(61) | 394(66) | 2(100) |
| LI | 44(61.11) | 41(64.06) | 55(68.75) | 58(63.04) | 131(66.16) | 249(68) | 41(59) | 8(50) | 8(89) | 5(83) | 18(47) | 117(64) | 208(66) | 5(83) |
| **When buying a food product, I prefer a long shelf life** |  |  |  |  |  |  |  |  |  |  |  |  |  |  |
| GEN | 92(65) | 134(70) | 153(66) | 101(55) | 270(63) | 527(62) | 99(72) | 34(55) | 40(68) | 6(75) | 37(73) | 385(66) | 363(61) | 2(100) |
| LI | 43(60) | 41(64) | 50(63) | 51(55) | 132(67) | 224(61) | 43(62) | 14(88) | 6(67) | 5(83) | 24(63) | 114(62) | 200(63) | 4(67) |
| **When buying a food product, I prefer a lower cost** |  |  |  |  |  |  |  |  |  |  |  |  |  |  |
| GEN | 94(67) | 149(78) | 154(66) | 131(71) | 274(63) | 586(69) | 85(62) | 39(63) | 43(73) | 4(50) | 36(71) | 377(65) | 423(71) | 2(100) |
| LI | 55(76) | 53(83) | 58(73) | 63(68) | 146(74) | 272(74) | 49(71) | 9(56) | 7(78) | 6(100) | 32(84) | 139(76) | 231(73) | 6(100) |

Data presented as n(%)

## ***Supplementary Table 4: Frequency and percentage of barriers and facilitators to healthy eating selected in each sample and demographic category.***

|  | **Age Group (years)** | | | | | **Race and Ethnicity** | | | | | | **Gender** | | |
| --- | --- | --- | --- | --- | --- | --- | --- | --- | --- | --- | --- | --- | --- | --- |
|  | **18-24** | **25-34** | **35-44** | **44-55** | **55+** | **White** | **Black** | **Hispanic** | **Asian** | **Multiple** | **Other** | **Male** | **Female** | **Non-Binary** |
| **Barriers to selecting healthy foods** |  |  |  |  |  |  |  |  |  |  |  |  |  |  |
| Price is too high |  |  |  |  |  |  |  |  |  |  |  |  |  |  |
| GEN | 68(48) | 120(63) | 139(60) | 113(61) | 188(44) | 460(54) | 57(41) | 35(56) | 33(56) | 3(38) | 34(67) | 259(44) | 367(61) | 2(100) |
| LI | 45(63) | 44(69) | 56(70) | 71(77) | 138(70) | 270(74) | 33(48) | 11(69) | 5(56) | 6(100) | 28(74) | 121(66) | 228(72) | 4(67) |
| Does not taste as good |  |  |  |  |  |  |  |  |  |  |  |  |  |  |
| GEN | 37(26) | 47(24) | 55(24) | 45(24) | 119(28) | 224(26) | 29(21) | 15(24) | 17(29) | 2(25) | 12(24) | 164(28) | 139(23) | 0(0) |
| LI | 23(32) | 12(19) | 17(21) | 20(22) | 50(25) | 92(25) | 11(16) | 4(25) | 3(33) | 1(17) | 11(29) | 43(23) | 75(24) | 4(67) |
| Shelf life too short |  |  |  |  |  |  |  |  |  |  |  |  |  |  |
| GEN | 34(24) | 48(25) | 47(20) | 26(14) | 46(11) | 143(17) | 26(19) | 8(13) | 14(24) | 0(0) | 7(14) | 98(17) | 102(17) | 1(50) |
| LI | 14(19) | 10(16) | 10(13) | 16(17) | 19(10) | 49(13) | 11(16) | 1(6) | 3(33) | 0(0) | 5(13) | 24(13) | 44(14) | 1(17) |
| Preparation time too long |  |  |  |  |  |  |  |  |  |  |  |  |  |  |
| GEN | 26(18) | 45(23) | 47(20) | 16(9) | 58(13) | 134(16) | 16(12) | 10(16) | 16(27) | 2(25) | 11(22) | 78(13) | 112(19) | 2(100) |
| LI | 8(11) | 6(9) | 11(14) | 14(15) | 18(9) | 43(12) | 8(12) | 1(6) | 2(22) | 1(17) | 2(5) | 15(8) | 42(13) | 0(0) |
| Not as available |  |  |  |  |  |  |  |  |  |  |  |  |  |  |
| GEN | 26(18) | 32(17) | 38(16) | 19(10) | 33(8) | 102(12) | 20(14) | 6(10) | 5(8) | 1(13) | 11(22) | 81(14) | 67(11) | 0(0) |
| LI | 15(21) | 13(20) | 15(19) | 11(12) | 30(15) | 57(16) | 11(16) | 4(25) | 4(44) | 2(33) | 6(16) | 35(19) | 48(15) | 1(17) |
| Serving size too small |  |  |  |  |  |  |  |  |  |  |  |  |  |  |
| GEN | 30(21) | 43(22) | 42(18) | 14(8) | 29(7) | 104(12) | 26(19) | 7(11) | 9(15) | 0(0) | 8(16) | 86(15) | 72(12) | 0(0) |
| LI | 14(19) | 5(8) | 8(10) | 10(11) | 12(6) | 29(8) | 12(17) | 1(6) | 1(11) | 0(0) | 6(16) | 25(14) | 22(7) | 2(33) |
| Lack of right cooking tools |  |  |  |  |  |  |  |  |  |  |  |  |  |  |
| GEN | 26(18) | 28(15) | 22(9) | 15(8) | 16(4) | 74(9) | 15(11) | 4(6) | 4(7) | 0(0) | 8(16) | 54(9) | 53(9) | 0(0) |
| LI | 13(18) | 11(17) | 9(11) | 13(14) | 13(7) | 45(12) | 9(13) | 0(0) | 1(11) | 0(0) | 4(11) | 23(13) | 36(11) | 0(0) |
| Nothing |  |  |  |  |  |  |  |  |  |  |  |  |  |  |
| GEN | 25(18) | 19(10) | 39(17) | 39(21) | 166(38) | 215(25) | 37(27) | 12(19) | 13(22) | 3(38) | 6(12) | 159(27) | 129(22) | 0(0) |
| LI | 9(13) | 7(11) | 11(14) | 15(16) | 35(18) | 50(14) | 14(20) | 3(19) | 2(22) | 0(0) | 8(21) | 34(18) | 43(14) | 0(0) |
| Other |  |  |  |  |  |  |  |  |  |  |  |  |  |  |
| GEN | 2(1) | 5(3) | 4(2) | 3(2) | 7(2) | 18(2) | 0(0) | 1(2) | 0(0) | 0(0) | 2(4) | 6(1) | 14(2) | 1(50) |
| LI | 0(0) | 1(2) | 1(1) | 2(2) | 4(2) | 8(2) | 0(0) | 0(0) | 0(0) | 0(0) | 0(0) | 4(2) | 4(1) | 0(0) |
| **Supports to selecting healthy foods** |  |  |  |  |  |  |  |  |  |  |  |  |  |  |
| Preparing grocery lists in advance |  |  |  |  |  |  |  |  |  |  |  |  |  |  |
| GEN | 47(33) | 83(43) | 107(46) | 80(43) | 218(50) | 403(47) | 50(36) | 28(45) | 23(39) | 1(13) | 25(49) | 230(39) | 303(51) | 2(100) |
| LI | 24(33) | 22(34) | 26(33) | 32(35) | 87(44) | 150(41) | 19(28) | 3(19) | 1(11) | 2(33) | 16(42) | 51(28) | 137(43) | 3(50) |
| Good-tasting recipes |  |  |  |  |  |  |  |  |  |  |  |  |  |  |
| GEN | 48(34) | 74(39) | 100(43) | 81(44) | 211(49) | 391(46) | 53(38) | 20(32) | 25(42) | 4(50) | 18(35) | 227(39) | 287(48) | 0(0) |
| LI | 34(47) | 18(28) | 31(39) | 32(35) | 70(35) | 134(37) | 26(38) | 7(44) | 3(33) | 1(17) | 14(37) | 64(35) | 118(37) | 3(50) |
| Budget-friendly recipes |  |  |  |  |  |  |  |  |  |  |  |  |  |  |
| GEN | 52(37) | 85(44) | 89(38) | 64(35) | 123(28) | 303(35) | 45(33) | 22(35) | 22(37) | 1(13) | 17(33) | 157(27) | 256(43) | 0(0) |
| LI | 32(44) | 22(34) | 31(39) | 33(36) | 63(32) | 135(37) | 14(20) | 8(50) | 3(33) | 2(33) | 19(50) | 55(30) | 122(39) | 4(67) |
| Meal prepping in advance |  |  |  |  |  |  |  |  |  |  |  |  |  |  |
| GEN | 47(33) | 62(32) | 71(30) | 53(29) | 102(24) | 239(28) | 39(28) | 16(26) | 16(27) | 1(13) | 22(43) | 156(27) | 179(30) | 0(0) |
| LI | 20(28) | 14(22) | 18(23) | 13(14) | 32(16) | 72(20) | 9(13) | 5(31) | 2(22) | 0(0) | 9(24) | 26(14) | 67(21) | 4(67) |
| Watching cooking videos |  |  |  |  |  |  |  |  |  |  |  |  |  |  |
| GEN | 41(29) | 60(31) | 66(28) | 53(29) | 82(19) | 209(24) | 42(30) | 18(29) | 18(31) | 2(25) | 11(22) | 148(25) | 154(26) | 0(0) |
| LI | 32(44) | 17(27) | 19(24) | 23(25) | 29(15) | 81(22) | 17(25) | 8(50) | 2(22) | 2(33) | 10(26) | 42(23) | 75(24) | 3(50) |
| Access to the right cooking tools |  |  |  |  |  |  |  |  |  |  |  |  |  |  |
| GEN | 38(27) | 34(18) | 37(16) | 22(12) | 52(12) | 118(14) | 31(22) | 11(18) | 9(15) | 1(13) | 13(25) | 108(19) | 75(13) | 0(0) |
| LI | 12(17) | 7(11) | 11(14) | 11(12) | 16(8) | 43(12) | 6(9) | 1(6) | 2(22) | 0(0) | 5(13) | 23(13) | 34(11) | 0(0) |
| Following health blogs/influencers |  |  |  |  |  |  |  |  |  |  |  |  |  |  |
| GEN | 27(19) | 45(23) | 45(19) | 30(16) | 28(6) | 118(14) | 21(15) | 10(16) | 12(20) | 3(38) | 11(22) | 83(14) | 92(15) | 0(0) |
| LI | 9(13) | 6(9) | 12(15) | 4(4) | 8(4) | 30(8) | 5(7) | 1(6) | 1(11) | 0(0) | 2(5) | 16(9) | 22(7) | 1(17) |
| Recommendations from organizations |  |  |  |  |  |  |  |  |  |  |  |  |  |  |
| GEN | 29(21) | 20(10) | 30(13) | 18(10) | 31(7) | 76(9) | 21(15) | 9(15) | 8(14) | 3(38) | 10(20) | 69(12) | 59(10) | 0(0) |
| LI | 9(13) | 3(5) | 8(10) | 4(4) | 7(4) | 20(5) | 7(10) | 2(13) | 1(11) | 0(0) | 1(3) | 14(8) | 17(5) | 0(0) |
| Consulting a dietitian/nutritionist |  |  |  |  |  |  |  |  |  |  |  |  |  |  |
| GEN | 16(11) | 23(12) | 24(10) | 26(14) | 33(8) | 89(10) | 14(10) | 5(8) | 5(8) | 1(13) | 7(14) | 64(11) | 58(10) | 0(0) |
| LI | 8(11) | 2(3) | 7(9) | 8(9) | 8(4) | 21(6) | 6(9) | 4(25) | 0(0) | 0(0) | 2(5) | 12(7) | 20(6) | 1(17) |
| Nothing |  |  |  |  |  |  |  |  |  |  |  |  |  |  |
| GEN | 17(12) | 20(10) | 32(14) | 30(16) | 5(1) | 136(16) | 19(14) | 4(6) | 10(17) | 3(38) | 9(18) | 109(19) | 75(13) | 0(0) |
| LI | 6(8) | 17(27) | 17(21) | 26(28) | 61(31) | 95(26) | 17(25) | 3(19) | 2(22) | 2(33) | 7(18) | 53(29) | 73(23) | 1(17) |
| Other |  |  |  |  |  |  |  |  |  |  |  |  |  |  |
| GEN | 1(1) | 1(1) | 4(2) | 1(1) | 19(4) | 22(3) | 0(0) | 1(2) | 0(0) | 0(0) | 3(6) | 11(2) | 15(3) | 0(0) |
| LI | 2(3) | 0(0) | 2(3) | 2(2) | 3(2) | 6(2) | 1(1) | 0(0) | 0(0) | 0(0) | 2(5) | 6(3) | 3(1) | 0(0) |

Data presented as n(%)

## ***Supplementary Table 5: Frequency and percentage of barriers to practicing healthy eating behaviors in each sample and each demographic category.***

|  |  | **Age Group (years)** | | | | | **Race and Ethnicity** | | | | | | **Gender** | | |
| --- | --- | --- | --- | --- | --- | --- | --- | --- | --- | --- | --- | --- | --- | --- | --- |
| **Item** | **Sample** | **18-24** | **25-34** | **35-44** | **45-55** | **55+** | **White** | **Black** | **Hispanic** | **Asian** | **Multiple** | **Other** | **Male** | **Female** | **Non-Binary** |
| **How often do you choose whole grain over refined grain?** |  |  |  |  |  |  |  |  |  |  |  |  |  |  |  |
| Never | GEN | 2(1) | 3(2) | 8(3) | 3(2) | 16(4) | 27(3) | 2(1) | 0(0) | 2(3) | 0(0) | 1(2) | 19(3) | 13(2) | 0(0) |
|  | LI | 2(3) | 6(9) | 5(6) | 7(8) | 15(8) | 23(6) | 10(14) | 1(6) | 0(0) | 0(0) | 1(3) | 11(6) | 24(8) | 0(0) |
| Rarely | GEN | 13(9) | 27(14) | 22(10) | 27(15) | 56(13) | 101(12) | 19(14) | 10(16) | 6(10) | 0(0) | 9(18) | 66(11) | 79(13) | 0(0) |
|  | LI | 16(22) | 10(16) | 12(15) | 16(17) | 18(9) | 53(14) | 9(13) | 3(19) | 2(22) | 0(0) | 5(13) | 23(13) | 48(15) | 1(17) |
| Sometimes | GEN | 64(46) | 69(36) | 94(41) | 70(38) | 176(41) | 348(41) | 46(33) | 26(42) | 27(46) | 5(63) | 21(41) | 225(39) | 246(41) | 2(100) |
|  | LI | 32(44) | 29(45) | 32(40) | 48(52) | 92(47) | 174(47) | 29(42) | 5(31) | 3(33) | 3(50) | 19(50) | 84(46) | 145(46) | 4(67) |
| Most of the time | GEN | 40(29) | 60(31) | 73(32) | 56(31) | 144(33) | 278(33) | 38(28) | 21(34) | 20(34) | 2(25) | 14(27) | 180(31) | 193(32) | 0(0) |
|  | LI | 17(24) | 14(22) | 21(26) | 16(17) | 62(31) | 94(26) | 15(22) | 5(31) | 4(44) | 2(33) | 10(26) | 53(29) | 76(24) | 1(17) |
| Always | GEN | 19(14) | 32(17) | 34(15) | 26(14) | 38(9) | 100(12) | 33(24) | 5(8) | 4(7) | 1(13) | 6(12) | 86(15) | 63(11) | 0(0) |
|  | LI | 5(7) | 5(8) | 10(13) | 5(5) | 10(5) | 23(6) | 6(9) | 2(13) | 0(0) | 1(17) | 3(8) | 12(7) | 23(7) | 0(0) |
| **What prevents you from making at least half of your grains whole grains**? |  |  |  |  |  |  |  |  |  |  |  |  |  |  |  |
| Taste preferences | GEN | 21(15) | 36(19) | 49(21) | 31(17) | 93(22) | 169(20) | 26(19) | 7(11) | 13(22) | 1(13) | 14(27) | 109(19) | 120(20) | 1(50) |
|  | LI | 19(26) | 14(22) | 18(23) | 26(28) | 41(21) | 81(22) | 19(28) | 4(25) | 3(33) | 1(17) | 10(26) | 41(22) | 76(24) | 1(17) |
| Cost | GEN | 22(16) | 38(20) | 46(20) | 34(19) | 61(14) | 146(17) | 21(15) | 9(15) | 14(24) | 2(25) | 9(18) | 89(15) | 110(19) | 2(100) |
|  | LI | 18(25) | 20(31) | 19(24) | 20(22) | 50(25) | 99(27) | 12(17) | 2(13) | 3(33) | 2(33) | 9(24) | 38(21) | 86(27) | 3(50) |
| I do not understand the difference between whole and refined grains | GEN | 18(13) | 20(10) | 20(9) | 15(8) | 44(10) | 87(10) | 9(7) | 5(8) | 4(7) | 2(25) | 10(20) | 49(9) | 68(11) | 0(0) |
|  | LI | 16(22) | 7(11) | 7(9) | 14(15) | 22(11) | 48(13) | 8(12) | 3(19) | 1(11) | 1(17) | 5(13) | 16(9) | 48(15) | 2(33) |
| I did not know this was recommended | GEN | 18(13) | 17(9) | 24(10) | 13(7) | 51(12) | 88(10) | 10(7) | 9(15) | 9(15) | 1(13) | 6(12) | 62(11) | 61(10) | 0(0) |
|  | LI | 10(14) | 9(14) | 4(5) | 15(16) | 17(9) | 41(11) | 6(9) | 2(13) | 2(22) | 0(0) | 4(11) | 27(15) | 27(9) | 1(17) |
| I'm not sure how to use/prepare | GEN | 11(8) | 19(10) | 17(7) | 10(5) | 30(7) | 58(7) | 12(9) | 5(8) | 7(12) | 0(0) | 5(10) | 40(7) | 47(8) | 0(0) |
|  | LI | 7(10) | 9(14) | 5(6) | 8(9) | 10(5) | 26(7) | 9(13) | 0(0) | 0(0) | 0(0) | 4(11) | 11(6) | 28(9) | 0(0) |
| Dietary restrictions | GEN | 5(4) | 11(6) | 5(2) | 6(3) | 10(2) | 23(3) | 7(5) | 2(3) | 2(3) | 0(0) | 3(6) | 23(4) | 13(2) | 1(50) |
|  | LI | 4(6) | 0(0) | 2(3) | 3(3) | 4(2) | 7(2) | 5(7) | 0(0) | 0(0) | 0(0) | 1(3) | 6(3) | 7(2) | 0(0) |
| Cultural/Religious preferences | GEN | 6(4) | 7(4) | 3(1) | 0(0) | 0(0) | 5(1) | 4(3) | 1(2) | 1(2) | 0(0) | 5(10) | 11(2) | 4(1) | 1(50) |
|  | LI | 4(6) | 0(0) | 1(1) | 1(1) | 0(0) | 3(1) | 3(4) | 0(0) | 0(0) | 0(0) | 0(0) | 3(2) | 3(1) | 0(0) |
| I do not care/try | GEN | 18(13) | 16(8) | 17(7) | 10(5) | 39(9) | 79(9) | 5(4) | 5(8) | 3(5) | 2(25) | 6(12) | 58(10) | 42(7) | 0(0) |
|  | LI | 8(11) | 6(9) | 5(6) | 7(8) | 17(9) | 35(10) | 5(7) | 1(6) | 1(11) | 0(0) | 1(3) | 15(8) | 28(9) | 0(0) |
| I don't know/unsure | GEN | 9(7) | 5(3) | 6(3) | 10(5) | 29(7) | 42(5) | 7(5) | 4(6) | 4(7) | 0(0) | 2(4) | 31(5) | 28(5) | 0(0) |
|  | LI | 2(3) | 5(8) | 7(9) | 13(14) | 12(6) | 26(7) | 5(7) | 3(19) | 1(11) | 0(0) | 4(11) | 14(8) | 25(8) | 0(0) |
| Other | GEN | 0(0) | 0(0) | 1(0) | 4(2) | 7(2) | 11(1) | 0(0) | 0(0) | 0(0) | 1(13) | 0(0) | 4(1) | 8(1) | 0(0) |
|  | LI | 0(0) | 0(0) | 0(0) | 3(3) | 4(2) | 7(2) | 0(0) | 0(0) | 0(0) | 0(0) | 0(0) | 3(2) | 4(1) | 0(0) |
| **How often do you choose healthy instead of unhealthy sources of protein?** |  |  |  |  |  |  |  |  |  |  |  |  |  |  |  |
| Never | GEN | 4(3) | 5(3) | 8(3) | 3(2) | 4(1) | 18(2) | 2(1) | 0(0) | 2(3) | 0(0) | 2(4) | 12(2) | 12(2) | 0(0) |
|  | LI | 4(6) | 1(2) | 3(4) | 3(3) | 10(5) | 16(4) | 2(3) | 0(0) | 0(0) | 0(0) | 3(8) | 9(5) | 12(4) | 0(0) |
| Rarely | GEN | 8(6) | 11(6) | 14(6) | 10(5) | 34(8) | 57(7) | 8(6) | 3(5) | 3(5) | 0(0) | 6(12) | 46(8) | 31(5) | 0(0) |
|  | LI | 11(15) | 11(17) | 10(13) | 13(14) | 20(10) | 51(14) | 7(10) | 1(6) | 1(11) | 0(0) | 5(13) | 22(12) | 42(13) | 1(17) |
| Sometimes | GEN | 49(36) | 59(31) | 84(36) | 75(41) | 145(34) | 300(35) | 48(35) | 25(40) | 25(42) | 4(50) | 10(20) | 201(35) | 210(35) | 1(50) |
|  | LI | 28(39) | 31(48) | 39(49) | 42(46) | 73(37) | 151(41) | 35(51) | 6(38) | 4(44) | 2(33) | 15(39) | 78(43) | 133(42) | 2(33) |
| Most of the time | GEN | 53(38) | 80(42) | 94(41) | 70(38) | 206(48) | 369(43) | 51(37) | 23(37) | 28(47) | 4(50) | 28(55) | 223(39) | 280(47) | 0(0) |
|  | LI | 22(31) | 20(31) | 14(18) | 30(33) | 72(37) | 117(32) | 16(23) | 7(44) | 4(44) | 3(50) | 11(29) | 57(31) | 98(31) | 3(50) |
| Always | GEN | 24(17) | 36(19) | 31(13) | 24(13) | 41(10) | 110(13) | 29(21) | 11(18) | 1(2) | 0(0) | 5(10) | 94(16) | 61(10) | 1(50) |
|  | LI | 7(10) | 1(2) | 14(18) | 4(4) | 22(11) | 32(9) | 9(13) | 2(13) | 0(0) | 1(17) | 4(11) | 17(9) | 31(10) | 0(0) |
| **What prevents you from choosing healthy sources of protein?** |  |  |  |  |  |  |  |  |  |  |  |  |  |  |  |
| Cost | GEN | 17(12) | 32(17) | 56(24) | 44(24) | 58(13) | 152(18) | 21(15) | 11(18) | 11(19) | 2(25) | 10(20) | 94(16) | 113(19) | 0(0) |
|  | LI | 19(26) | 24(38) | 26(33) | 27(29) | 51(26) | 112(31) | 15(22) | 3(19) | 3(33) | 2(33) | 12(32) | 56(31) | 89(28) | 2(33) |
| Taste preferences | GEN | 19(14) | 31(16) | 50(22) | 34(19) | 91(21) | 169(20) | 17(12) | 14(23) | 15(25) | 0(0) | 10(20) | 119(21) | 106(18) | 0(0) |
|  | LI | 18(25) | 16(25) | 16(20) | 23(25) | 44(22) | 82(22) | 17(25) | 3(19) | 2(22) | 1(17) | 12(32) | 51(28) | 64(20) | 2(33) |
| I did not know this was recommended | GEN | 15(11) | 11(6) | 10(4) | 5(3) | 13(3) | 32(4) | 11(8) | 3(5) | 6(10) | 0(0) | 2(4) | 27(5) | 27(5) | 0(0) |
|  | LI | 2(3) | 6(9) | 4(5) | 3(3) | 5(3) | 15(4) | 1(1) | 2(13) | 0(0) | 0(0) | 2(5) | 11(6) | 9(3) | 0(0) |
| I'm not sure how to use/prepare | GEN | 7(5) | 7(4) | 10(4) | 3(2) | 10(2) | 25(3) | 6(4) | 0(0) | 5(8) | 0(0) | 1(2) | 18(3) | 19(3) | 0(0) |
|  | LI | 9(13) | 4(6) | 3(4) | 5(5) | 3(2) | 13(4) | 8(12) | 0(0) | 1(11) | 0(0) | 2(5) | 7(4) | 17(5) | 0(0) |
| Dietary restrictions | GEN | 5(4) | 5(3) | 5(2) | 5(3) | 8(2) | 19(2) | 6(4) | 1(2) | 1(2) | 0(0) | 1(2) | 15(3) | 13(2) | 0(0) |
|  | LI | 1(1) | 2(3) | 2(3) | 3(3) | 2(1) | 8(2) | 1(1) | 0(0) | 0(0) | 0(0) | 1(3) | 6(3) | 4(1) | 0(0) |
| Cultural/Religious preferences | GEN | 2(1) | 5(3) | 5(2) | 1(1) | 0(0) | 6(1) | 4(3) | 1(2) | 2(3) | 0(0) | 0(0) | 9(2) | 3(1) | 1(50) |
|  | LI | 2(3) | 1(2) | 1(1) | 1(1) | 0(0) | 1(0) | 2(3) | 1(6) | 0(0) | 0(0) | 1(3) | 3(2) | 2(1) | 0(0) |
| I do not understand which are healthy choices | GEN | 7(5) | 11(6) | 10(4) | 6(3) | 17(4) | 35(4) | 5(4) | 4(6) | 5(8) | 0(0) | 2(4) | 30(5) | 21(4) | 0(0) |
|  | LI | 6(8) | 2(3) | 10(13) | 12(13) | 15(8) | 36(10) | 5(7) | 0(0) | 1(11) | 0(0) | 3(8) | 14(8) | 30(9) | 1(17) |
| I do not care/try | GEN | 12(9) | 12(6) | 16(7) | 6(3) | 26(6) | 49(6) | 9(7) | 3(5) | 6(10) | 2(25) | 3(6) | 43(7) | 29(5) | 0(0) |
|  | LI | 5(7) | 5(8) | 7(9) | 8(9) | 20(10) | 37(10) | 5(7) | 1(6) | 0(0) | 0(0) | 2(5) | 15(8) | 30(9) | 0(0) |
| I don't know/unsure | GEN | 4(3) | 2(1) | 4(2) | 9(5) | 14(3) | 24(3) | 7(5) | 2(3) | 0(0) | 0(0) | 0(0) | 16(3) | 17(3) | 0(0) |
|  | LI | 3(4) | 4(6) | 4(5) | 9(10) | 8(4) | 20(5) | 4(6) | 1(6) | 1(11) | 0(0) | 2(5) | 10(5) | 18(6) | 0(0) |
| Other | GEN | 1(1) | 3(2) | 4(2) | 1(1) | 8(2) | 16(2) | 0(0) | 1(2) | 0(0) | 0(0) | 0(0) | 7(1) | 10(2) | 0(0) |
|  | LI | 0(0) | 0(0) | 1(1) | 1(1) | 3(2) | 5(1) | 0(0) | 0(0) | 0(0) | 0(0) | 0(0) | 3(2) | 2(1) | 0(0) |
| None of the above | GEN | 4(3) | 3(2) | 3(1) | 0(0) | 11(3) | 15(2) | 2(1) | 2(3) | 1(2) | 1(13) | 0(0) | 12(2) | 9(2) | 0(0) |
|  | LI | 2(3) | 2(3) | 0(0) | 5(5) | 2(1) | 6(2) | 3(4) | 0(0) | 0(0) | 0(0) | 2(5) | 6(3) | 5(2) | 0(0) |

Data presented as n(%)
